# *Legionella* in operational healthcare water systems: a case-based approach to investigation, interpretation, and control

**DOI:** 10.1016/j.infpip.2026.100580

**Published:** 2026-08-12

**Authors:** Saied Ali, Anne-Marie Dolan, Susan Lapthorne, Maeve Doyle

**Affiliations:** a Department of Clinical Microbiology, Royal College of Surgeons in Ireland, Dublin, Ireland; b Department of Clinical Microbiology, Beaumont Hospital, Dublin, Ireland; c Department of Clinical Microbiology, Cork University Hospital, Cork, Ireland; d Department of Clinical Microbiology, University Hospital Waterford, Waterford, Ireland

**Keywords:** Infection prevention and control, *Legionella*, Legionnaires’ disease, Outbreak investigation, Water safety group, Water systems

## Abstract

*Legionella* species are environmental Gram-negative bacteria capable of colonising artificial water systems and causing Legionnaires’ disease, a severe form of atypical pneumonia. Healthcare facilities are particularly vulnerable to transmission because complex building water systems can support bacterial growth and aerosol generation, exposing highly susceptible patient populations.

This article uses a case-based scenario to illustrate the investigation of suspected healthcare-associated Legionnaires’ disease on a haematology ward. The recognition of clustered pneumonia cases prompted multi-disciplinary investigation involving infection prevention and control teams, clinical microbiologists, and estates engineers. The article outlines the regulatory and technical frameworks that underpin *Legionella* risk management in healthcare facilities and the role of Water Safety Groups in co-ordinating environmental investigation.

Key elements of laboratory diagnostics are described, as well as practical aspects of environmental sampling, including the use of preflush and postflush samples to determine whether contamination is localised to outlets or reflects colonisation of the wider water distribution system. Interpretation of quantitative results is discussed alongside common engineering findings such as inadequate temperature control, stagnation, and redundant pipework. The article also outlines immediate precautionary measures and longer-term remediation strategies used to control *Legionella* in hospital water systems.

Effective prevention of healthcare-associated Legionnaires’ disease requires close collaboration between infection prevention and control teams, microbiologists, and estates professionals to ensure safe design, monitoring, and management of healthcare water systems.

## What is *Legionella* and why is it important in healthcare-associated infection prevention and control?

*Legionella* species are aerobic Gram-negative bacilli that occur naturally in freshwater environments, but are also capable of colonising artificial water systems [1]. Within aquatic environments, *Legionella* species survive and multiply within free-living amoebae and other protozoa. This ability contributes to environmental persistence and is closely linked to the organism’s capacity to survive and replicate within human alveolar macrophages following inhalation of contaminated aerosols [1]. The genus comprises more than sixty recognised species, although approximately 90% of human disease is caused by *Legionella pneumophila*. Serogroup 1 accounts for approximately 80% of cases, while serogroups 2–15 are also recognised human pathogens and together account for most of the remaining *L. pneumophila* infections. Other *Legionella* species, including *L. longbeachae*, *L. micdadei*, *L. bozemanii*, and *L. dumoffii*, account for a relatively small proportion of reported cases, particularly among immunocompromised individuals and in specific epidemiological settings [2]. *L. longbeachae* is more commonly associated with soil and commercial potting compost than aquatic environments and is reported more frequently in Australia and New Zealand. *L. micdadei* (historically referred to as the Pittsburgh pneumonia agent) is an important opportunistic pathogen in immunocompromised patients and may appear weakly acid-fast on staining [2].

Infection typically presents as either Legionnaires’ disease, a severe atypical pneumonia, or the milder, self-limiting Pontiac fever. These clinical entities differ in both severity and incubation period, with Legionnaires’ disease developing 2–10 days after exposure (and up to 14 days in some cases), whereas Pontiac fever has a much shorter onset, usually within a few hours to 2–3 days. Legionnaires’ disease is characterised by fever and respiratory symptoms, frequently accompanied by extra-pulmonary features such as diarrhoea or neurological manifestations including confusion. Laboratory abnormalities, particularly hyponatraemia and mild hepatic dysfunction, may further raise clinical suspicion [1,3,4].

Transmission occurs primarily through inhalation of aerosolised contaminated water or, less commonly, aspiration of contaminated drinking water. Person-to-person transmission is extremely rare, and the epidemiology of *Legionella* infection is therefore closely linked to environmental reservoirs [5]. Travel-associated disease related to contaminated water systems in hotels, resorts, and cruise ships has been linked to approximately 10–20% of cases, with countries such as Italy, Spain, and France accounting for a substantial proportion [6].

Healthcare-associated Legionnaires’ disease (HA-LD) carries a higher case-fatality rate than community-acquired infection, reflecting the vulnerability of hospitalised populations [7]. In healthcare settings, transmission is typically linked to colonisation of building water systems, where *Legionella* may persist within plumbing infrastructure and subsequently be dispersed through aerosol-generating outlets, such as showers, taps, or other water fixtures.

## What regulatory and technical guidance frameworks underpin *Legionella* risk management in healthcare facilities?

Control of *Legionella* in healthcare facilities is governed through a combination of occupational health legislation and technical guidance for the management of building water systems, which define the legal duties of organisations to assess, monitor, and control the risk of *Legionella* proliferation within water infrastructure. The principal documents are summarised in Table I.

**Table I:** Regulatory and technical frameworks guiding *Legionella* risk management in healthcare water systems

Framework	Type of document	Key relevance to *Legionella* control
Health and Safety at Work etc. Act 1974 (HSWA) [8]	Primary legislation	Establishes the overarching duty of employers to protect employees, patients, and visitors from health risks arising from workplace activities, including risks associated with building water systems such as exposure to *Legionella*.
Control of Substances Hazardous to Health Regulations 2002 (COSHH) [9]	Statutory regulations	Requires organisations to assess and control exposure to hazardous biological agents, including *Legionella*, through risk assessment, implementation of preventive measures, and documentation of control procedures.
Approved Code of Practice L8: Legionnaires’ disease – The control of *Legionella* bacteria in water systems (Health and Safety Executive; HSE ACOP L8) [10]	Approved Code of Practice	Provides practical guidance for compliance with *Legionella* control legislation, including appointment of a responsible person, development of a written scheme of control, and monitoring of water system management.
Legionnaires’ disease: Technical guidance, Part 2 – The control of *Legionella* bacteria in hot- and cold-water systems (Health and Safety Executive; HSG274 Part 2) [11]	Technical guidance	Provides operational guidance on management of hot- and cold-water systems, including temperature control, flushing regimes, system design considerations, and maintenance practices that reduce conditions favourable for *Legionella* growth.
Health Technical Memorandum 04-01: Safe water in healthcare premises (HTM 04-01) [12]	Healthcare infrastructure guidance	Provides detailed recommendations for the design, operation, and governance of water systems in healthcare settings, including the establishment of multi-disciplinary Water Safety Groups and structured water safety plans.
Water Safety Plan framework (World Health Organization; WHO) [13]	International public health guidance	Promotes a risk-based approach to managing water systems from source to point-of-use, supporting identification and control of hazards such as *Legionella* within healthcare water systems.
Standard 188: Legionellosis – Risk management for building water systems (American Society of Heating, Refrigerating and Air-Conditioning Engineers; ASHRAE Standard 188) [14]	Engineering standard	Provides internationally recognised requirements for building water management programmes aimed at preventing *Legionella* growth and transmission.
Developing a Water Management Program to reduce *Legionella* growth and spread in buildings (Centers for Disease Control and Prevention; CDC) [15]	Public health guidance	Provides practical guidance for developing water management programmes, including hazard assessment, monitoring strategies, and outbreak response in healthcare and other buildings.
Legionnaires’ disease surveillance and outbreak investigation guidance (European Centre for Disease Prevention and Control; ECDC) [6]	European public health guidance	Supports surveillance, case definitions, and co-ordinated investigation of Legionnaires’ disease across Europe, including guidance for outbreak detection and response.

**Table II:** Interpretation of *Legionella* concentrations in water samples [12]

*Legionella* concentration	Interpretation	Recommended response
Not detected or ≤ 100 cfu/L	Satisfactory; No significant contamination detected or very low-level colonisation	Continue routine monitoring and maintain existing control measures. Any detection should prompt review of the local risk assessment and WSP. In areas serving highly susceptible patients, consider interim mitigation measures (e.g. restricting use of affected outlets, point-of-use filters, or enhanced flushing) while repeat sampling and investigation are undertaken, where appropriate.
>100–1000 cfu/L	Undesirable; Evidence of colonisation	If only a small proportion of samples are positive, repeat sampling should be performed and control measures reviewed. If multiple outlets are positive, the system may be colonised at low level and a formal review of water system controls and risk assessment should be undertaken. Consider interim mitigation measures for outlets serving high-risk patient populations pending investigation and repeat culture results.
>1000 cfu/L	Unsatisfactory; Significant colonisation of the water system	Immediate investigation required. Review engineering controls and risk assessment urgently and implement remedial actions such as system disinfection. Appropriate interim risk-reduction measures, including restricting patient exposure to affected outlets and/or installation of point-of-use filters where indicated, should be implemented while remediation and repeat sampling are undertaken.

cfu/L, colony-forming units per litre; WSP, water safety plan.

**Table III:** Interpretation of preflush and postflush sampling results [12]

Result pattern	Likely interpretation	Typical action
Pre-flush > Postflush	Contamination likely localised to the outlet (e.g. tap, aerator, shower head, flexible hose)	Clean, disinfect, or replace outlet components; remove flexible hoses; review flushing and outlet maintenance
Preflush ≈ Postflush (both high)	Suggestive of colonisation of the wider water distribution system	Investigate system-wide control measures including temperature management, circulation, and disinfection; engineering remediation likely required
Preflush ˂ Postflush	Contamination arising from upstream pipework or the wider water distribution system	Investigate stagnation points, system temperatures, circulation, and storage tanks; further environmental sampling and engineering review required
Preflush and postflush both low or negative	No significant colonisation detected	Continue routine surveillance and maintain existing control measures

Rather than replacing existing guidance documents, this article is intended as a practical educational resource that illustrates the application of current best practice through a case-based approach for clinicians, microbiologists, infection prevention and control (IPC) practitioners, and members of Water Safety Groups (WSGs). The recommendations presented reflect current national guidance and standard practice at the time of writing. Readers are encouraged to consult the latest versions of relevant national guidance documents, as recommendations may evolve.

Clinical scenario: Three patients on a haematology ward developed severe pneumonia over a two-week period, two of whom required intensive care support. All had been hospitalised for more than five days prior to symptom onset. Routine bacterial cultures were negative and clinicians raised the possibility of *Legionella* infection. How should *Legionella* infection be investigated in the clinical microbiology laboratory?

The most commonly used rapid diagnostic test is urinary antigen detection; however, currently available assays only detect *Legionella pneumophila* serogroup 1 and may therefore miss infections caused by other species or serogroups [3]. False-positive results are uncommon but have been reported in specific circumstances, including persistent antigenuria following recent *Legionella* infection, cross-reactivity with other bacterial infections such as *Streptococcus pneumoniae* or *Pseudomonas aeruginosa*, and interference from factors such as rheumatoid factor or exposure to rabbit antilymphocyte serum. As the detected antigen is heat stable, repeat testing after boiling the urine specimen at 100°C for 5 min, followed by centrifugation and testing of the cooled supernatant, may be used in selected cases to help confirm true positivity [16]. Molecular methods, particularly polymerase chain reaction (PCR) performed on respiratory specimens such as sputum or bronchoalveolar lavage, offer improved sensitivity and broader detection of *Legionella* species [3].

Culture remains the reference method and is particularly important for epidemiological investigation, as recovery of clinical isolates permits comparison with environmental strains during an outbreak. *Legionella* species are fastidious and require specialised media such as buffered charcoal yeast extract (BCYE) agar supplemented with L-cysteine and iron salts [17]. Colonies may become visible after 3–5 days of incubation at 35–37°C; however, culture plates should be incubated for 7–10 days before being reported as negative to allow recovery of slow-growing isolates. Colonies are typically convex with a characteristic glistening, “ground-glass” appearance, while pigmentation may vary from grey-white to green or purple depending on the species and the age of the culture. Biochemically, *Legionella* species are oxidase variable, catalase positive, and non-lactose fermenters [17].

## When should healthcare-associated Legionnaires’ disease be suspected and what initial steps should be taken?

HA-LD should be suspected when pneumonia develops more than 48 h after hospital admission, particularly among higher-risk patients such as older adults (especially those aged >50 years), smokers, and individuals with chronic lung disease or immunocompromising conditions; and where cases cluster within the same clinical area or share exposure to hospital water sources [1,7]. Identification of two or more cases within a defined period should prompt urgent investigation and early involvement of IPC teams. Initial actions include confirming case definitions, notifying IPC services, and activating the hospital WSG [6,12,18].

The WSG typically includes estates engineers, IPC practitioners, consultant microbiologists, facilities and risk management staff, and senior hospital management, with specialist contractors involved where required. It oversees water safety plans (WSPs), environmental risk assessment, and monitoring of hospital water systems. Where multiple cases are identified, an outbreak or incident control team should be convened. This multi-disciplinary team usually includes IPC staff, microbiology or infectious diseases specialists, nursing representatives, estates personnel, hospital management, occupational health, communications teams, and public health authorities, and co-ordinates the investigation, environmental testing, implementation of control measures, and communication with relevant stakeholders [12].

Beyond co-ordinating the response to suspected or confirmed cases, the WSG also has responsibility for overseeing routine verification of the WSP to ensure that control measures remain effective over time. This may include periodic environmental monitoring of representative sites identified through local risk assessment, such as hot water storage vessels, sentinel outlets, and other points within the distribution system that are considered most informative for assessing overall system performance. The purpose of this surveillance is to verify that the water system remains under control rather than to investigate individual clinical cases.

In contrast, when HA-LD is suspected or confirmed, environmental sampling should be directed by the epidemiological investigation and focus on water outlets and associated sections of the distribution system to which affected patients were exposed. The investigation should consider each patient’s accommodation, ward movements, and potential water exposures during the incubation period, as patients may have acquired infection in clinical areas other than the ward where disease was recognised. Consequently, environmental sampling may need to include previously occupied wards, other areas of the hospital, or additional locations identified through the epidemiological investigation. The sampling strategy should be expanded further where engineering findings or microbiological results suggest more widespread colonisation.

## Following identification of two HA-LD cases, the WSG recommends a review of *Legionella* control measures and environmental sampling of the hospital water system serving the affected ward. Samples are collected from multiple outlets including hand-wash basin taps and showers. How is environmental sampling for *Legionella* performed in healthcare settings?

Samples are collected in sterile containers containing an appropriate neutralising agent (e.g. sodium thiosulfate) to inactivate residual disinfectants that may otherwise reduce the recovery of *Legionella* during transport. Where practicable, sampling should be undertaken early in the day or following periods of low water use to maximise the likelihood of detecting *Legionella* that may have accumulated during stagnation. Approximately 1 L of water is collected from each sampling point, allowing concentration of organisms by membrane filtration prior to culture [11,12].

Sampling is usually performed in two stages. A preflush sample is collected immediately after the outlet is opened fully, before water is allowed to run, representing water within the outlet and adjacent pipework. A postflush sample is collected after the water has run for two minutes, representing water from the wider distribution system [11,12]. Water temperatures should also be measured and recorded at the time of sampling. Measurement of preflush and postflush temperatures provides an important assessment of temperature control at the outlet and assists in interpreting microbiological findings and identifying engineering deficiencies. Where investigation extends beyond individual outlets, temperatures should also be verified at hot- and cold-water storage units and other sentinel points within the distribution system to assess overall system performance.

Where point-of-use filters are fitted, consideration should be given to removing the filter if the objective is to assess the underlying water distribution system rather than the performance of the filter itself. Similarly, outlets should not be disinfected prior to sample collection, including before postflush sampling, as this may alter the microbiological findings and reduce recovery of organisms present under normal operating conditions.

Sampling sites are selected based on potential exposure risk and the configuration of the water system. In addition to high-risk outlets such as showers, hand-wash basin taps, thermostatic mixing valve (TMV) outlets, and non-TMV outlets (e.g. those in sluice rooms, waste-handling areas, and cleaners’ cupboards), together with hot water storage tanks (calorifiers) and cold-water storage tanks, it is essential that the system is adequately represented through sampling of sentinel outlets, defined by the plumbing layout, including proximal and distal points within the distribution network and outlets on affected wards [11,12].

Samples are concentrated by filtration, and the concentrate is typically divided into untreated and acid- or heat-treated aliquots prior to culture. Each aliquot is cultured in parallel on BCYE agar and a selective medium, such as glycine–vancomycin–polymyxin B–cycloheximide (GVPC) agar. While *Legionella* spp. grow on BCYE agar, environmental water samples frequently contain substantial background microbial flora; therefore, selective media are used alongside BCYE to suppress competing organisms and improve recovery of *Legionella* spp. Results are reported as colony-forming units per litre (cfu/L) or, where a full 1-L sample cannot be obtained, as cfu per volume of water sampled [11,12,19]. Where *Legionella* is recovered from environmental samples, representative isolates should be retained, where feasible, to permit sequence-based typing (SBT) or whole-genome sequencing (WGS) if subsequent epidemiological investigation or comparison with clinical isolates becomes necessary.

Although aerosol sampling has been investigated as a potential method for assessing exposure risk from contaminated water systems, current guidance does not recommend its routine use during healthcare investigations and its role remains primarily research-based. This reflects the lack of standardised sampling methodologies, variable organism recovery, and uncertain correlation between aerosol detection and clinical risk. Consequently, environmental investigation continues to rely on targeted water sampling interpreted alongside epidemiological and engineering findings.

**Environmental sampling from the haematology ward water system produced the following results:**

**Table:** 

Outlet	Preflush (cfu/L)	Postflush (cfu/L)
Shower room	**1500**	**1400**
Patient sink	**250**	**30**
Patient sink (bed 4)	**<10**	**<10**

Cfu, colony-forming units.

## How should *Legionella* water sampling results be interpreted?

Interpretation of environmental results involves two main considerations: the absolute concentration of *Legionella* detected and the relationship between preflush and postflush samples (Table II, Table III).

Interpretation should also take into account the *Legionella* species identified, where available. Although routine species differentiation is not required for all environmental samples, identification of *L. pneumophila*, particularly serogroup 1, provides important clinical context as it accounts for the majority of HA-LD. Other *Legionella* species, including *L. anisa*, *L. longbeachae*, *L. micdadei*, *L. bozemanii*, and *L. dumoffii*, are recognised opportunistic pathogens, particularly in immunocompromised individuals, although they are identified less frequently in clinical disease. In addition, the detection of *L. anisa* in hospital water systems should not be regarded as benign. Evidence suggests that it may act as a surrogate marker for undetected *L. pneumophila* colonisation, potentially masking low-level *L. pneumophila* populations during culture. Consequently, detection of *L. anisa* should prompt careful risk assessment and consideration of further investigation, particularly in healthcare settings serving vulnerable patient populations [20]. Interpretation should therefore integrate the species identified, quantitative culture results, the patient population served, and the overall environmental risk assessment when determining the need for remedial action.

Environmental culture results should always be interpreted within the wider clinical and engineering context. In healthcare settings, particularly those caring for highly susceptible patients, management decisions should not rely solely on quantitative thresholds. Because repeat culture results may take several days to become available, detection of *Legionella* should prompt a timely multi-disciplinary risk assessment and consideration of proportionate interim control measures, including restricting patient exposure to affected outlets, installing point-of-use filters where appropriate, temporarily removing affected outlets from clinical use while enhanced flushing and remedial engineering interventions are undertaken, and providing alternative water sources where necessary until investigations are complete.

In addition to bacterial concentration, a comparison of preflush and postflush samples helps determine whether contamination is localised to the outlet or originates from the wider water distribution system.

In this scenario, the shower outlet demonstrates persistently high counts in both samples, suggesting colonisation of the upstream water system rather than contamination confined to the outlet. In contrast, the reduction in counts after flushing in the sink outlet suggests contamination limited to the tap or associated fittings.

However, it is important to bear in mind that although persistently elevated counts in both samples are generally suggestive of more widespread system colonisation, heavy biofilm accumulation within outlets or immediately adjacent pipework may continue to release *Legionella* during flushing, resulting in persistently high counts despite contamination remaining relatively localised. Consequently, sampling results should be interpreted alongside the overall distribution of positive sites, engineering findings, and knowledge of the water distribution system. Where contamination appears confined to distal pipework or outlet components, targeted cleaning, disinfection, or replacement of the affected fittings should be considered before concluding that more extensive system-wide remediation is required.

## Further investigation of the water system by the WSG and estates engineers identified deficiencies in temperature control within the hot water system. Hot water outlet temperatures were recorded at approximately 45–48°C. What control measures should be implemented following detection of *Legionella* in a hospital water system?

While engineering remediation is undertaken, immediate precautionary measures should be implemented to minimise patient exposure to potentially contaminated water sources as previously described. This may include restricting the use of affected outlets, providing alternative water sources for patient hygiene where required, installing point-of-use filters, enhanced flushing of infrequently used outlets, and increased clinical surveillance for additional cases of *Legionella* infection [1,12].

This should be accompanied by a systematic review of the water distribution system led by the WSG. This includes assessment of pipework design and condition, with particular attention to identifying and removing dead legs, redundant pipework, and other features that promote stagnation. Engineering review should also identify factors that may compromise temperature control, including damaged or inadequate pipe insulation (lagging), poor hot water circulation or system balancing, infrequently used outlets, deficiencies in storage vessels or TMV configuration, and elevated ambient temperatures within plant rooms or clinical areas, particularly in highly glazed buildings, which may impair maintenance of cold-water temperatures.

In the scenario described, persistently elevated counts across outlets, together with suboptimal water temperatures, indicated likely system-wide colonisation and failure of existing control measures.

Recommended parameters include hot water storage temperatures ≥60°C and distribution temperatures ≥55°C. At outlets not fitted with TMVs, hot water should typically reach ≥50–55°C within one minute. Where TMVs are installed, compliance with hot water temperature targets should be assessed upstream of the mixing valve, as the mixed outlet temperature is intentionally reduced to minimise scalding risk. Cold water should remain <20°C within two minutes [12]. In our case, remediation therefore focused on restoring temperature control.

Following implementation of corrective measures, repeat environmental sampling should generally be undertaken two to seven days after disinfection and refilling of the system to confirm that *Legionella* counts have decreased and that control has been restored. Sampling immediately after disinfection should be avoided, as it may produce falsely negative results. Further sampling should be performed at intervals determined by the risk assessment until satisfactory and sustained control has been demonstrated; failure of improvement would necessitate additional remediation.

Thermal disinfection (thermal shock) involves raising hot water temperatures to approximately 70–80°C and flushing outlets sequentially, providing rapid reduction of bacterial counts, although the effect may be temporary if underlying system deficiencies are not corrected. Hyperchlorination, in which elevated concentrations of free chlorine are introduced into the water system, can also achieve short-term control but may be corrosive to pipework. Longer-term control strategies include continuous chemical disinfection, such as chlorine dioxide dosing, which can penetrate biofilms but requires specialist equipment and monitoring. Copper–silver ionisation systems provide another option for sustained control in large water systems through the release of antimicrobial metal ions, although installation and ongoing monitoring are required [11,12].

Recurrent or persistent contamination despite appropriate remediation may require more extensive engineering investigation, specialist environmental assessment, and additional exploratory testing tailored to the specific characteristics of the water system.

## Recent literature on *Legionella* control in healthcare environments

Molecular typing has become increasingly important in the investigation of Legionnaires’ disease outbreaks. For many years, *Legionella pneumophila* isolates were characterised using SBT, a method that analyses the nucleotide sequences of seven housekeeping genes to assign isolates to specific sequence types. SBT has been widely used through international surveillance networks to compare clinical and environmental isolates during outbreak investigations [21].

More recently, WGS has emerged as a powerful tool for investigating *Legionella* transmission. By analysing the entire bacterial genome, WGS provides far greater resolution than traditional typing methods and allows more precise assessment of the relatedness between clinical and environmental isolates. Several studies have demonstrated the use of WGS in confirming links between hospital water systems and patient infections, while also distinguishing unrelated environmental strains that may coexist within the same plumbing network [22].

Genomic studies have also highlighted the substantial diversity of *Legionella* populations within building water systems. Multiple strains may be present within a single water source, and persistent colonisation can occur over prolonged periods as organisms adapt to biofilm-associated environments [23]. These findings emphasise that genomic data should be interpreted alongside epidemiological information and environmental investigation.

## Take home messages

• *Legionella* infection should be considered when severe pneumonia arises during hospitalisation, particularly where cases occur in clusters or when exposure to hospital water systems is possible.
• Diagnosis relies on urinary antigen testing, PCR of respiratory specimens, and culture on specialised media such as BCYE and GVPC agar for epidemiological investigation; however, urinary antigen assays primarily detect *Legionella pneumophila* serogroup 1 and may therefore miss infections caused by other species or serogroups.
• Environmental investigation includes targeted water sampling with preflush and postflush samples to distinguish outlet contamination from wider system colonisation.
• Temperature control remains central to prevention, with hot water typically maintained at ≥60°C in storage and ≥50–55°C at outlets, and cold water <20°C.
• Effective control requires close collaboration between microbiology, infection prevention teams, estates engineers, and the WSG.
• *Legionella* prevention in healthcare settings is guided by a number of regulatory and engineering frameworks, including the Health and Safety at Work Act, Control of Substances Hazardous to Health Regulations 2002 (COSHH), HSE Approved Code of Practice L8, HSG274, and HTM 04-01, which together underpin water safety governance and risk management.
• Remediation strategies must address both microbial contamination and underlying engineering deficiencies, including stagnation, dead legs, and inadequate temperature control.

## CRediT authorship contribution statement

**Saied Ali:** Writing – review & editing, Writing – original draft, Resources, Project administration, Methodology, Investigation, Formal analysis, Data curation, Conceptualization. **Anne-Marie Dolan:** Writing – review & editing, Investigation, Formal analysis. **Susan Lapthorne:** Writing – review & editing, Writing – original draft, Resources, Investigation. **Maeve Doyle:** Writing – review & editing, Validation, Supervision, Project administration.

## Ethical approval

Ethical approval was not required for this work. This manuscript is a narrative educational review incorporating a hypothetical case scenario for instructional purposes and does not involve original research, human participants, animal subjects, or identifiable patient data.

## Declaration of generative AI and AI-assisted technologies in the writing process

The authors declare that no generative AI or AI-assisted technologies were used in the preparation of this manuscript.

## Funding sources

None to declare.

## Conflict of interest statement

None to declare.
